# Effects of Survival Processing on Item and Context Memory: Enhanced Memory for Survival-Relevant Details

**DOI:** 10.3389/fpsyg.2020.02244

**Published:** 2020-09-11

**Authors:** Zoie R. Meyers, Matthew P. McCurdy, Ryan C. Leach, Ayanna K. Thomas, Eric D. Leshikar

**Affiliations:** ^1^Department of Psychology, University of Illinois at Chicago, Chicago, IL, United States; ^2^Department of Psychology, Tufts University, Medford, MA, United States

**Keywords:** survival processing, item memory, context memory, source memory, adaptive memory

## Abstract

Due to the natural selection pressure, certain aspects of memory may have been selected to give humans a survival advantage. Research has demonstrated that processing information for survival relevance leads to better item memory (i.e., the content of information) compared to control conditions. The current study investigates the effects of survival processing on context memory (i.e., memory for peripheral episodic details) and item memory to better understand when the survival processing memory advantage emerges. In this study, participants studied pictures of objects in either a survival or moving (control) condition. Objects were presented in either a plausible color, for example, a red apple, or in an implausible color, such as a green pie. We chose this color plausibility manipulation because color is a detail that conveys information about the fitness (and other diagnostic information) about an item. After studying items, participants made item memory judgments (did you see this item before?) and two context memory judgments: color context (in which color did you see this item?) and source context (in which condition did you see this item?). Results indicated better item memory for materials processed in the survival relative to moving condition. Critically, for color context, there was a condition by plausibility interaction, where memory was best for plausibly colored items in the survival processing condition. There was no difference, however, in source context memory between the survival and moving conditions. These results suggest the survival processing memory advantage extends to contextual details that particularly reflect the survival utility of items such as color.

## Introduction

A theoretical framework suggests that due to natural selection, certain aspects of human memory have been tuned to give people a survival and reproductive advantage (Nairne et al., 2007). This functional-evolutionary perspective of memory suggests that ancestral selection pressures have shaped our memory systems to better remember certain survival-relevant information such as the location of food or information about predators (Nairne et al., 2007; Nairne, 2010). Prior investigations on survival processing often require participants to study items under either survival (i.e., “imagine yourself stranded in a foreign land”) versus some comparison control conditions (e.g., “imagine yourself moving to a foreign land”) and then are tested on their memory for these studied items. A growing body of evidence has demonstrated that information processed for survival relevance is better remembered than information processed under various control conditions (Kang et al., 2008; Nairne et al., 2008; Otgaar et al., 2010; Burns et al., 2011; Scofield et al., 2018; though see Butler et al., 2009; Kroneisen and Erdfelder, 2011), suggesting that survival processing is a robust memory phenomenon.

Although prior research has shown a survival processing advantage for item memory (Scofield et al., 2018), less is known about the effects of survival processing on context memory (i.e., memory for peripheral episodic details during study). The limited studies on survival processing effects on context have produced mixed results, with some evidence in support of context memory improvements compared to controls (Nairne et al., 2012; Clark and Bruno, 2016; Kroneisen and Bell, 2018; Misirlisoy et al., 2019; Zhang et al., 2020), whereas others have found no such benefits (Bröder et al., 2011; Savine et al., 2011; Nairne et al., 2015; Hou and Liu, 2019). In a study reporting a survival processing context memory advantage, participants were shown items in various locations on a computer screen and asked to process them in either a survival or scavenger hunt condition (Nairne et al., 2012). In both survival and scavenger conditions, participants were asked to consider their starting position at the center of the screen and then rate how difficult or easy it would be to collect each item. Results indicated substantially better location context memory for items in the survival compared to scavenger (control) condition. By contrast, in another study of context memory, participants processed faces in either a survival condition (choose people to help you hunt to survive) or a non-survival control condition (choose people to help you win a hunting contest) and then tested whether they could remember the condition under which each face was studied (did you rate this face in the survival/hunting condition?; i.e., source context memory) before a final recall test (Experiment 2; Savine et al., 2011). Context memory results showed no difference in memory for the source context recognition task between survival and hunting (control). Taken together, these mixed findings potentially imply that memory for only certain contextual details is enhanced under survival processing conditions, which makes sense given that remembering certain contextual details such as whether food looks ready for consumption or where a food source or predator is located could enhance survival. Thus, it may be that context memory is especially enhanced for certain details that convey survival-relevant information about studied items. One detail that may tap into the survival relevance of an object is color. For example, a healthy-looking red apple would likely confer more survival benefit than an apple that appears off-colored. This idea is supported by findings that color relays diagnostic information about the quality of food such as fruits and vegetables (Barrett et al., 2010; Prokop and Fančovičová, 2014). In the animal kingdom, abundant evidence suggests that color is an essential cue in food-seeking behavior (Logue, 1980; Laska and Metzker, 1998; Raine and Chittka, 2008). Even further, in humans, work shows that even when food is perfectly edible, but colored in an unusual way, this leads to changes in how appealing that food is, and further, how the food is even perceived in terms of taste (Garber et al., 2000; Spence, 2016), which further suggests the adaptive importance of this feature (color). In addition to food, color might also give meaningful information about predators such as remembering the exact coloring of a particularly vicious predator, or it might convey health information about a potential predator (such as a predator with a sickly-looking coat). In this study, we examined the survival processing advantage for two different contextual features to better understand whether a context memory advantage might emerge for only some contextual details and not others. Specifically, we investigated source context memory, which does not seem to reliably induce a survival processing advantage (Nairne et al., 2010; Experiments 1, 2, and 3; Savine et al., 2011; Nairne et al., 2015; Hou and Liu, 2019; though see Kroneisen and Bell, 2018; Misirlisoy et al., 2019; Zhang et al., 2020, for studies that did report a source context memory advantage) and color context, which is a detail that conveys diagnostic information about studied items.

The current study investigates the effects of survival processing on item and context memory. We were particularly interested in examining whether memory for contextual details might improve for a detail that strongly conveys fitness of an item, such as color. In this experiment, half of the items were shown in a plausible color (i.e., high plausibility), whereas the other half were shown in an implausible color (i.e., low plausibility). Participants studied colored objects in survival and moving scenarios. At test, item memory and memory for two contextual details were assessed: color context, where participants reported in which color an object originally appeared (red, blue, or green), and source context, where participants reported in which condition an object was initially studied (survival and moving). We made three predictions in this investigation. First, for item memory, we predicted that materials processed in the survival condition would be better remembered than in the moving (control) condition consistent with prior work (Nairne et al., 2007; Scofield et al., 2018). Second, for color context memory, we expected to see an especially large context survival processing advantage for highly plausible items such as a red apple compared to lower plausible items (e.g., green pie). Such results would converge with other findings that contextual details that signal survival-relevant information (such as location) may be especially prone to exhibit a survival context memory advantage (Nairne et al., 2012; Clark and Bruno, 2016). In contrast, since a survival processing source context memory advantage has been less reliable with some studies finding an advantage (Kroneisen and Bell, 2018; Misirlisoy et al., 2019; Zhang et al., 2020), but others not (Nairne et al., 2010; Savine et al., 2011; Nairne et al., 2015; Hou and Liu, 2019), we did not make strong predictions for source context memory.

## Materials and Methods

### Participants

Twenty-seven adults (eight males and 19 females), recruited from the University of Illinois at Chicago, participated in this experiment. Because we used a recognition memory test in this experiment, we chose to base a power analysis on a recent survival processing paper that used a recognition memory task (Misirlisoy et al., 2019). A power analysis using G^*^Power (Faul et al., 2007) estimated 25 participants would give us 80% power to detect differences across conditions. All participants gave their informed, written consent required by the institutional review board at the University of Illinois at Chicago. Participants were paid or received course credit for participating.

### Stimuli

Stimuli consisted of 72 common objects colored red, blue, or green. The objects were from three categories: food (e.g., apple, avocado, and pie), animals (e.g., bear, rabbit, and rat), and inanimate objects (e.g., soap, jacket, and cup; see Appendix Table A1 for the percentage of stimuli from these categories). The images were taken from Hemera stock images and online. Half of the objects were plausibly colored, for example, a red apple. The other half of the objects were not plausibly colored, for example, a green pie. The plausibility of all objects was determined in a pilot study conducted prior to the current study where participants reported how plausible it was to see an item (e.g., apple) in a particular color (i.e., red, blue, and green)^1^. Across participants, items were counterbalanced to appear in the survival and moving conditions at encoding as well as novel items at retrieval. For example, a red apple would appear in the survival condition, the moving condition, or as a lure (in grayscale) at retrieval across participants.

### Procedure

There were two phases of the experiment, an intentional^2^ study phase (encoding) and a test phase (retrieval). Before starting the encoding phase, participants were given both verbal and written training on task instructions for study and test phases of the experiment, which included practice of both experimental phases (12 practice trials for study; 12 practice trials for retrieval). After training, participants completed the encoding phase of the experiment.

**Figure 1:**
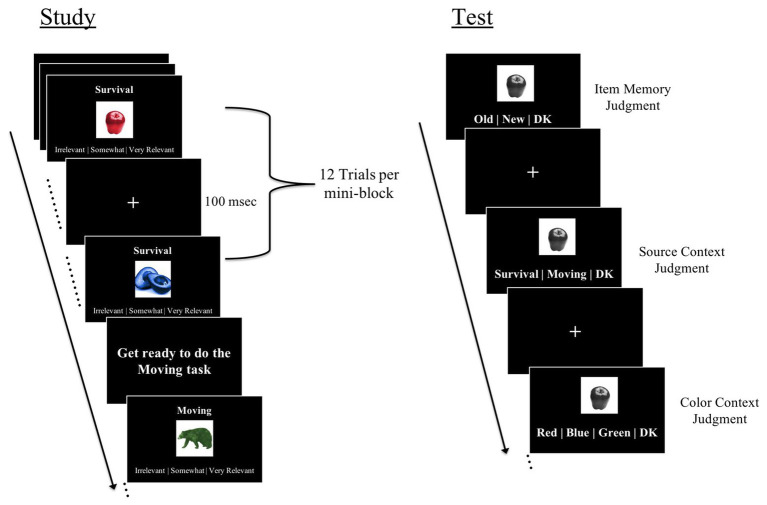
Trial schematic for several study phase trials and a single test phase trial.

At encoding, participants were shown a total of 48 objects. Participants studied these objects in one of two encoding conditions: a survival condition and a moving (control) condition. For items presented in the survival processing condition, participants were instructed to rate how relevant each object would be to help them survive in a remote, foreign land without any basic supplies^3^. For items presented in the moving (control) condition, participants were instructed to judge how relevant each object would be to help them move to, and get settled in, a foreign land^4^. As in past work (Nairne et al., 2007; Butler et al., 2009; Otgaar et al., 2010; Bröder et al., 2011; Kroneisen and Erdfelder, 2011; Bell et al., 2013; Kroneisen and Bell, 2018), we chose the moving task to serve as the control condition because this task induces schematic processing similar to survival processing task, but without the survival component. On each encoding trial, participants were shown one object colored red, blue, or green (Figure 1). The object was presented in the center of the screen with the task instruction (survival or moving) written above the object and the rating scale (irrelevant/somewhat relevant/very relevant) written below. Each trial was self-paced and was followed by a fixation of 100 ms. Trials were presented in four alternating mini-blocks of the survival task and moving task (12 items per mini-block; half plausible and half implausible). Within a mini-block, plausible and implausible items were randomly intermixed.

Immediately following encoding, participants completed the retrieval phase of the experiment. A total of 72 objects were presented. Of those, 48 were old (studied at encoding), and 24 were new (unstudied) items^5^. Because we were interested in memory for the color associated with studied items, all items were shown in grayscale during retrieval. For each item, participants made three self-paced recognition decisions, corresponding to item, source context, and color context memory, respectively (Figure 1). First, participants were shown an object in the center of the screen and instructed to judge whether the object was old (seen at encoding), new, or if they “do not know” (i.e., item memory judgment). Second, participants judged whether they studied the item in the survival or moving (control) condition at encoding or whether they did not know (i.e., source context memory judgment). Third, participants were asked to judge whether the item appeared in red, blue, or green color at encoding or whether they did not know (i.e., color context memory judgment). Participants were instructed to respond “do not know” if they were unsure of their decision, which reduces guessing, as done in past work (Duarte et al., 2008; Leshikar and Duarte, 2012, 2014; Leshikar et al., 2015). Since “do not know” responses were used to reduce guessing, these trials were not included in our primary memory estimates. Participants made all three judgments for all trials (regardless of whether they endorsed items as old or new). For items endorsed as novel, participants were instructed to use the “do not know” response for the source and color context judgments.

## Results

In this section, we first report encoding phase responses, followed by analyses of the retrieval phase data^6^. At encoding, for *high-plausible* items, participants endorsed 43% of the items as very relevant, 23% as somewhat relevant, and 34% as irrelevant in the *survival* condition, and in the *moving* (control) condition, they endorsed 19% of the items as very relevant, 31% as somewhat relevant, and 50% as irrelevant. For *low-plausible* items, participants endorsed 43% of the items as very relevant, 29% as somewhat relevant, and 28% as irrelevant in the *survival* condition, and in the *moving* (control) condition, they endorsed 11% of the items as very relevant, 34% as somewhat relevant, and 55% as irrelevant. To compare these encoding relevance ratings, we examined the relationship between encoding condition (survival or moving) and the relevancy responses for both high-plausibility and low-plausibility items separately. The chi-square tests of independence for both the high-plausible items, *χ*^2^(2, *N* = 648) = 41.39, *p* < 0.001, and low-plausible items, *χ*^2^(2, *N* = 648) = 92.47, *p* < 0.001, revealed there was a significant difference in encoding ratings across the encoding conditions. Finding relevancy rating differences across tasks, especially higher relevance rates for survival compared to control conditions, is in line with several past investigations (Nairne et al., 2008; Röer et al., 2013; Kroneisen and Bell, 2018); however, because we found a significant difference between relevancy responses and encoding condition, we also conducted three separate 2 (condition: survival and moving) × 3 (encoding response: very relevant, somewhat relevant, and irrelevant) ANOVAs on item and context (source and color) memory hit rates to determine if encoding responses were linked to subsequent memory for those items. For item memory, this analysis revealed a marginal main effect for condition, *F*(1, 19) = 3.92, *p* = 0.06, *η*^2^ = 0.172, that showed that survival items (*M* = 0.91, *SD* = 0.08) were better remembered than moving items (*M* = 0.87, *SD* = 0.11), regardless of how participants responded to those items. Importantly, the main effect for response and condition by response interaction were not significant (*F*s < 1.81, *p*s > 0.19), suggesting that memory performance was not strongly affected by how participants responded to the relevance of items at encoding. For both color and source context, we found no main effects of condition, *F*s < 1.58, *p*s > 0.19, or relevancy, *F*s < 1.36, *p*s > 0.27, and no interaction, *F*s < 2.57, *p*s > 0.09. Overall, these analyses suggest that although there were differences in relevancy ratings across encoding conditions, these differences did not seem to strongly influence later memory performance^7^.

**Figure 2:**
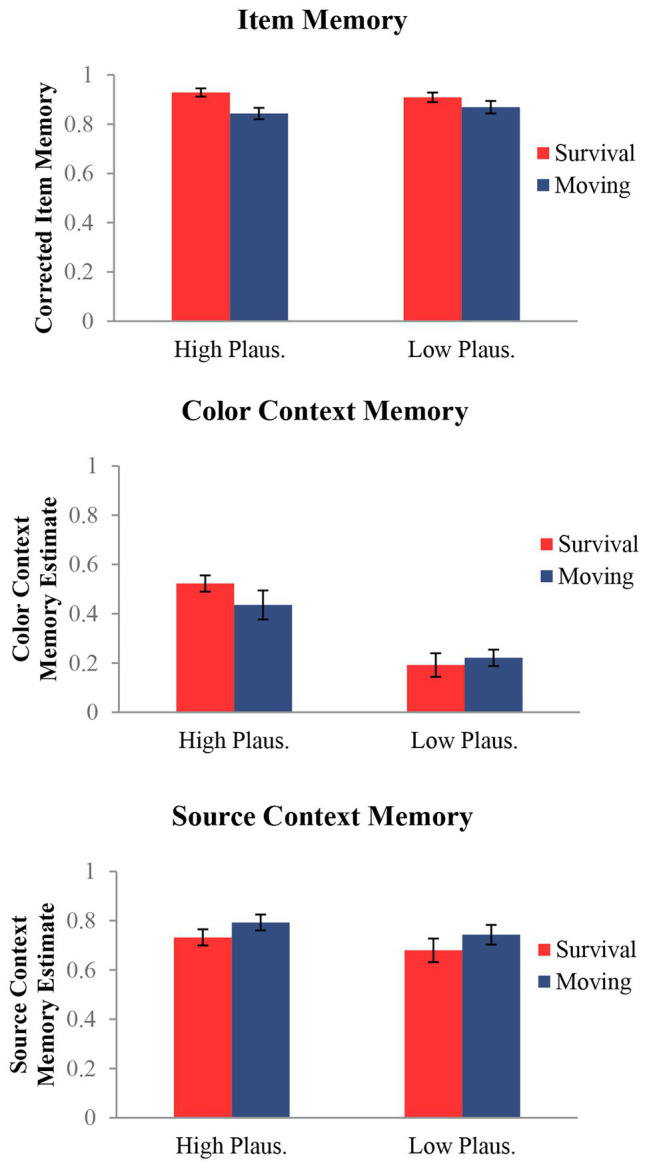
Measures of memory for item and both context details (color and source) as functions of condition and plausibility. Results indicated a survival processing effect for item memory consistent with prior work. For color context, results showed a plausibility by condition interaction driven by better memory for highly plausible colored items in the survival processing condition. There were no effects for source context.

Item and context memory responses (color and source) are presented in Table 1 as a function of encoding condition. To assess memory performance, we calculated the rates of corrected item, color context, and source context. For all three memory measures (item, color context, and source context), we conducted 2 (condition: survival and moving) × 2 (plausibility: high and low) repeated-measures ANOVAs (Figure 2).

**Table 1:** Means and standard deviations (in parentheses) of mean raw responses for item, source, and color recognition.

High-plausibility objects											
Item recognition				Source context				Color context			
Task	Old	New	Do not know	Task	Survival	Moving	Do not know	Task	Correct	Incorrect	Do not know
Survival	0.93 (0.09)	0.05 (0.08)	0.02 (0.04)	Survival	0.70 (0.17)	0.20 (0.16)	0.10 (0.11)	Survival	0.49 (0.27)	0.22 (0.22)	0.29 (0.28)
Moving	0.87 (0.12)	0.11 (0.09)	0.02 (0.06)	Moving	0.12 (0.13)	0.71 (0.18)	0.17 (0.15)	Moving	0.37 (0.25)	0.25 (0.23)	0.38 (0.27)
**Low-plausibility objects**											
**Item recognition**				**Source context**				**Color context**			
Task	Old	New	Do not know	Task	Survival	Moving	Do not know	Task	Correct	Incorrect	Do not know
Survival	0.91 (0.09)	0.08 (0.09)	0.01 (0.03)	Survival	0.63 (0.23)	0.23 (0.21)	0.14 (0.15)	Survival	0.19 (0.16)	0.37 (0.21)	0.44 (0.28)
Moving	0.88 (0.13)	0.09 (0.11)	0.03 (0.05)	Moving	0.12 (0.13)	0.69 (0.22)	0.19 (0.16)	Moving	0.21 (0.21)	0.27 (0.17)	0.52 (0.29)
**Novel objects**											
**Item recognition**				**Source context**				**Color context**			
	FA	CR	Do not know			FA	CR			FA	CR
	0.01 (0.03)	0.97 (0.03)	0.02 (0.03)			0.06 (0.06)	0.94 (0.10)			0.04 (0.09)	0.96 (0.06)

FA, false alarm; CR, correct rejection.

Corrected item memory was calculated by subtracting the average false alarm rate in each condition (e.g., new items endorsed as having appeared in the survival or moving condition, respectively) from the average item hit rate (a studied item endorsed as old) as done by past work (Zhang et al., 2019). This procedure allowed us to calculate corrected measures of item memory using hits and false alarm rates for each respective encoding condition (instead of a single pooled false alarm rate for all new items). Results of the item recognition analysis revealed a main effect of condition, *F*(1, 26) = 11.09, *p* = 0.003, *η*^2^ = 0.30. This effect was driven by better memory for items processed in the survival (*M* = 0.92, *SD* = 0.07) compared to the moving condition (*M* = 0.86, *SD* = 0.11) consistent with previous studies (Nairne et al., 2007; Scofield et al., 2018). There was no main effect for plausibility or interaction, *F*s < 2.54, *p*s > 0.12^8^.

Color context memory estimates for both encoding conditions (survival, moving) were calculated as the proportion of color hits (correct color recognition) out of item hits (correct item recognition), which removes the influence of item memory on the context memory measures (Murnane and Bayen, 1996), as we have done before (Leshikar et al., 2014, 2016; Leshikar and Gutchess, 2015). Results of the color context analysis revealed no main effect of condition, *F*(1, 26) = 1.17, *p* = 0.288, *η*^2^ = 0.04. There was, however, a main effect of plausibility, *F*(1, 26) = 23.98, *p* =< 0.001, *η*^2^ = 0.48, reflecting better color memory for high-plausibility items (*M* = 0.48, *SD* = 0.28) compared to low (*M* = 0.21, *SD* = 0.18). Critically, this main effect was accompanied by a condition by plausibility interaction, *F*(1, 26) = 8.05, *p* = 0.009, *η*^2^ = 0.24, which showed that high-plausibility items were remembered better than low-plausibility items for both conditions, but that this difference (high > low plausibility) was greater in the survival compared to the moving condition. This is consistent with our prediction that memory for details that reflect the survival utility of items would be especially enhanced under survival processing.

Source context memory estimates for both encoding conditions (survival, moving) were calculated using an analogous procedure as color context: we calculated the proportion of source hits (correct source recognition) out of item hits (correct item recognition). Results of the source context recognition analysis showed no significant results, *F*s < 3.63, *p*s > 0.07, *η*^2^ < 0.12, suggesting that survival processing did not have a statistically significant effect on source context memory. These findings are consistent with prior work probing memory for source context (Nairne et al., 2010; Hou and Liu, 2019; Experiments 1, 2, and 3; Savine et al., 2011; Nairne et al., 2015)^9^.

## Discussion

In this experiment, we examined the survival processing memory benefit for item memory and multiple contextual details. We were particularly interested in context memory for different episodic details to investigate whether survival processing might improve context memory for features that reflect the survival utility of items. We report two main findings. First, we found a survival item memory advantage, where items processed in the survival condition were better remembered than the moving (control) condition, consistent with a growing body of work (Nairne et al., 2007; Kang et al., 2008; Otgaar et al., 2010; Scofield et al., 2018). Second, we found that color context, but not source, showed a survival processing memory benefit when compared against the moving (control) condition. Overall, these data support the idea that the survival processing memory benefit extends to some contextual details that are especially reflective of the survival utility of studied items.

In this study, we found evidence that item memory was better for materials processed in the survival condition relative to control, which is consistent with prior work (Nairne et al., 2007). We found this effect using an intentional memory design (participants knew about the upcoming memory test), which means our finding extends past work that has most often used incidental memory designs (where participants do not know about the memory test). This is meaningful because this suggests that regardless of intent to try to remember information, processing items for survival enhances memory. Survival processing frequently produces item memory benefits under a variety of conditions (e.g., compared against various control tasks; Nairne et al., 2007; Kang et al., 2008; with pictorial stimuli, Otgaar and Smeets, 2010; in adults and children; Otgaar et al., 2010), and our findings further extend this work by demonstrating a survival item memory effect for pictorial stimuli under intentional encoding instructions. Although we found an item memory survival processing effect, there was no effect of plausibility on item memory, suggesting that plausible coloring of items had no influence on subsequent item recognition decisions. Indeed, it is possible that items presented in implausible colors (e.g., green bear) could have induced bizarreness effects (McDaniel and Einstein, 1986), making those items more distinctive and hence more memorable, but we did not see evidence of such effects in our data. Overall, data from this experiment support the idea that survival processing is a powerful memory strategy that enhances item memory.

The primary focus of this investigation was to examine the effect of survival processing on memory for multiple contextual details. As others have argued (Bröder et al., 2011), if survival processing truly reflects an adaptive use of memory, then it seems likely that memory for at least some details pertinent to survival, such as the location of a food source (Nairne et al., 2012; Clark and Bruno, 2016), or color of objects, should show a survival context memory advantage. Our color context memory findings are consistent with this rationale. Specifically, our color context memory results revealed a condition by plausibility interaction resulting from better memory for plausibly colored items (e.g., red apple) relative to implausibly colored items (e.g., green pie) that was larger in the survival than moving condition. It may be that color provides useful and important diagnostic information about food for consumption (Barrett et al., 2010; Prokop and Fančovičová, 2014) or the voracity of a threat (predator), and thus memory might be especially attuned to these details, which may reflect an adaptive use of memory. Interestingly, although we found that relevancy rating were not related to color context memory, it is worth noting that relevancy ratings were high (items endorsed as relevant or somewhat relevant) in the survival processing condition for both high- and low-plausibility items. What this could mean is that participants were not fully attending to this detail (color) in the survival processing condition (e.g., they were only attending to the object to make decisions, and not the color). We see this possibility as less likely however because, if this were true, we would not expect to see color context memory differ between the high- and low-plausibility items in the survival processing condition as we did. Overall, finding better context memory for high-plausibility items compared to low-plausibility items when thinking about items for their survival utility is consistent with other work that has found context memory improvement for certain details that tap into the survival relevance of items. Our findings, taken together with prior work (Experiment 2; Nairne et al., 2012; Clark and Bruno, 2016), suggest that certain details may be especially memorable under survival processing conditions.

Thus far, we have discussed the item memory and color context survival processing advantage we observed in these data, but an important question is whether these effects may in part be attributable to known memory mechanisms. Past work on survival processing effects have focused on understanding potential memory mechanisms (e.g., “proximate” mechanisms) that might partially account for the survival processing memory advantage. Although some accounts are not well-supported by the literature (such as an interactive imagery mechanism; Kroneisen et al., 2013), others show promise in partially explaining survival processing effects. One such account is that survival processing, relative to non-survival control conditions, induces both enhanced item-specific processing (processing of items in a way that makes that item sufficiently distinguishable from other items on a memory test), as well as enhanced relational processing of studied items (processing of relationship between the item and other features such as list membership or other contextual details; Burns et al., 2011). We observed evidence for both mechanisms (enhance item-specific and relational processing) in these data. Past work has shown that recognition memory tests of the sort we used in this investigation are especially sensitive to enhanced item-specific processing (Einstein and Hunt, 1980; Burns, 2006), and thus it may be that survival processing induced enhanced item-specific processing relative to the moving condition, giving rise to the item memory effect we observed. In addition to enhanced item-specific processing, this account (enhanced item-specific and relational processing) further suggests that survival processing induces enhanced relational processing. Our color context finding provides some evidence in line with this idea. Although some of the past work on enhanced relational processing has focused on how survival processing enhances memory across trials (for example, across lists of items that are related), work in another memory domain (generation effect) has shown that enhanced relational processing leads to increased performance for context memory (Marsh et al., 2001; McCurdy et al., 2017, 2019, 2020). Thus, in the present data, it may be that survival processing induced enhanced relational processing between the item and its color that was especially pronounced for the plausibly colored items under survival processing conditions. Taken together, our item and color context findings are consistent with the idea that survival processing enhances both item-specific and relational processing relative to control conditions, which contributes to our understanding of survival processing memory effects observed in the literature.

In contrast to color context, we found no evidence of a survival processing memory advantage for source context. Although some have argued that a survival processing effect in source memory *should* be robust (Bröder et al., 2011), many studies, including the current study, have failed to observe such a memory advantage (Nairne et al., 2010; Savine et al., 2011; Nairne et al., 2015, Hou and Liu, 2019) versus those studies that have (Kroneisen and Bell, 2018; Misirlisoy et al., 2019; Zhang et al., 2020). Given these mixed findings, it may be that there is a source memory survival processing advantage, but that it is small and thus harder to detect. Indeed, a careful look through past work investigating source memory effects generally shows numerically better source memory in the survival processing condition compared to control conditions, even when the difference does not reach significance (Savine et al., 2011; Hou and Liu, 2019)^10^. Consistent with the idea that the survival processing effect may be small (and unreliable), Misirlisoy et al. (2019) conducted four experiments examining source memory survival processing effects. In one of the four experiments (Experiment 1a), there was no survival source memory benefit. A second experiment (Experiment 2b) showed conflicting results based on different analyses. Thus, only two of the four experiments fully supported a source memory survival processing effect, which is consistent with the idea that the survival processing effect for source context exists but is not reliable. It is curious (perhaps even surprising) that a survival source context memory advantage is not a common finding across the literature. After all, it is easy to argue that the sheer act of thinking about the survival utility of items should in itself be sufficient to induce a survival processing advantage. Instead, it seems that simply thinking about survival utility is not sufficient in itself to afford a reliable source context memory advantage. It is therefore possible that an item must be seen as relevant to survival before a source context memory advantage might be observed. Such a possibility is consistent with “congruity” effects in past survival processing investigations that show that the memory advantage is enhanced only for those items most relevant to a survival context (Butler et al., 2009). It is worth stating though that our data are not consistent with this possibility because we observed no significant relationship between relevancy ratings at encoding and context memory performance. Future work is needed to better understand why a source context memory effect is not a reliable finding.

In this experiment, we found evidence of a survival processing memory advantage. It may be that this memory phenomenon is adaptive because remembering certain types of survival-relevant information, such as the location of a food source, could allow one to plan for survival in the future. Indeed, work in other domains suggests that memory for information about future events tend to be better remembered than information that does not involve future planning (Klein et al., 2010). This in turn is consistent with other work suggesting that people use the contents of memory in order to think about the future (Szpunar et al., 2007; Szpunar, 2010; Frankenstein et al., 2020). In the current study, it may be that better memory for highly plausible items, such as a red apple, may be useful for future planning, such as deciding when food is ready to be consumed. Altogether, these findings suggest that details that pertain to planning for the future, such as one’s future survival, are prioritized in memory, which may have been shaped in our evolutionary past. Finding ways to improve memory is an important pursuit (Rogers et al., 1977; Butler and Roediger, 2007; Leshikar et al., 2012, 2017; Matzen et al., 2015; Leach et al., 2018; McCurdy et al., 2020), and this work contributes to a wider body of research investigating how memory might be enhanced.

Although we found evidence of an item and color context survival processing memory advantage, it is worth discussing some limitations to this work in detail and how future work might address these limitations. First, our source context memory measure was yoked to the encoding condition since participants were asked to report in which encoding condition items were studied. Although this is a common procedure in the memory literature (Johnson et al., 1993) as well as in survival processing investigations (Savine et al., 2011; Kroneisen and Bell, 2018; Hou and Liu, 2019; Zhang et al., 2020), future work on source context memory effects under survival processing conditions might use a different source task that is not related to the encoding task such as list membership (in which list was this word studied?, list 1, list 2, etc.) or voice source (in which voice was this word spoken? voice 1, voice 2, etc.). Such work could extend the source context memory findings presented in this paper. Second, our color manipulation involved high versus low plausibly colored items. Because we used low-plausible items that were by definition not common, it may be that such items might have induced phenomenon like bizarreness effects, making those items more distinctive, and thus more memorable. Although any possible memory effects induced by this manipulation were equivalent in both conditions (and thus less likely to influence our main finding), future work investigating color context effects might use different materials, such as items that are all plausibly colored, but that still convey fitness. Third, as demonstrated in the *Introduction*, color gives important diagnostic information about food and predators, but we also included a small set of objects that does not fit these categories (e.g., inanimate objects), and thus for these items, color may be less diagnostic. Future work, therefore, might use only food (or only animals) to extend the survival processing color context memory advantage we observed in these data. Fourth, although analyses showed that plausibility did not affect relevancy ratings across conditions, nor did relevancy ratings interact with condition in any of our memory measures, it is still possible that memory could have been influenced as a function of condition, plausibility, and relevancy ratings. Given that we did not design this experiment to look at all three factors simultaneously, future work in this domain should use designs that can do so, such as using more stimuli per condition.

## Conclusion

Overall, we found evidence of an item memory benefit under the survival processing condition relative to a moving (control) condition consistent with previous work. We also found evidence that survival processing supports context memory for certain details, but only those that strongly convey information relevant for survival (such as the color of an item). These findings offer some clarity on previous studies that have produced mixed context memory results. This enhanced memory for item and context for materials processed for survival supports the idea that due to ancestral selection pressures, human memory systems may have been shaped to better remember information that is survival-relevant. Altogether, these findings support the idea that survival processing is a potent memory phenomenon that produces item memory benefits, as well as context memory benefits, but only for some contextual details, which may reflect an adaptive function of memory.

## Data Availability Statement

The raw data supporting the conclusions of this article will be made available by the authors, without undue reservation.

## Ethics Statement

The studies involving human participants were reviewed and approved by University of Illinois at Chicago IRB. The participants provided their written informed consent to participate in this study.

## Author Contributions

ZM, AT, and EL were involved in the conception of the research idea. ZM collected the data. ZM, MM, RL, and EL were involved in analysis of the data. ZM, MM, RL, and EL wrote and edited the manuscript. We thank the Research Open Access Publishing (ROAAP) Fund of the University of Illinois at Chicago for financial support towards the open access publishing fee for this article. All authors contributed to the article and approved the submitted version.

### Conflict of Interest

The authors declare that the research was conducted in the absence of any commercial or financial relationships that could be construed as a potential conflict of interest.

## Figures and Tables

**Table A1:** Distribution of stimuli included in this experiment.

Type of object	
	Percent of stimuli
Food	0.50
Animals	0.21
Inanimate objects	0.29

## Footnotes

^1^In that pilot, an independent sample of participants used a plausibility scale ranging from 1 (not plausible) to 9 (very plausible). The average plausibility rating for the high-plausibility items was *M* = 8.92, *SD* = 0.099. The average plausibility rating for the low-plausibility items was *M* = 1.06, *SD* = 0.079. We used the high-plausibility items to create items of higher “fitness” (e.g., red apple) and the low-plausibility items to create those of low “fitness” (e.g., green pie).

^2^Although much of the work on survival processing has involved incidental encoding, we chose to make this study intentional to expand the generalizability of survival processing memory effect findings to intentional encoding conditions.

^3^The instructions for the survival task were: “In this task, we would like you to imagine that you are stranded in the grasslands of a foreign land without any basic materials. Over the next few months, you will need to find food and water, and protect yourself from predators. Please rate how relevant each item would be to you in this survival situation.”

^4^The instructions for the moving task were: “In this task, we would like you to imagine that you are moving to a new home in a foreign land. Over the next few months, you will need to purchase a home and transport your belongings. Please rate how relevant each item would be to you in this moving situation.”

^5^Since our primary interest in this investigation was on source context memory, we chose to use lure items that would be balanced for the source context decision. That is, our use of 48 old items (24 survival and 24 moving) and 24 novel items means that a participant would be equally endorsing all three response options for the source context decision. If we had used half old and new items at recognition (48 each), this would mean that participants would not be using all three source context response options equivalently.

^6^We designed this experiment to examine how encoding condition and plausibility influenced the survival processing effect. We did not, however, design this experiment with theoretical interest in the factor of initial relevancy rating. This is important because in these data, we cannot examine memory as a function of condition, plausibility, and relevancy all in the same analysis because we would not have sufficient trials for all participants to do so.

^7^We also examined response times (RTs) for the relevancy decision at encoding since it is possible that longer response times could reflect important processing differences across tasks that might have interacted with plausibility (for the high- versus low-plausibility items). To do this, we conducted a 2 (condition: survival and moving) by 2 (plausibility) ANOVA on mean RTs. Only the main effect of plausibility was significant, *F*(1, 26) = 18.81, *p* < 0.001, *η*^2^ = 0.14. The plausibility effect was driven by faster RTs to the plausible items (*M* = 2,274 ms, *SD* = 121 ms) compared to the implausible items (*M* = 2,085 ms, *SD* = 114 ms). No other effects were significant, *F*s < 4.09, *p*s > 0.05. Importantly, although we did see an effect of plausibility on RTs, it is worth noting that the interaction was not significant, which suggests that the possible influence of RTs on subsequent memory was likely similar across conditions and thus not likely to influence our main interpretations of the memory data. As we note in footnote 6, it was not possible for us to analyze RTs as a function of condition, plausibility, and relevancy in the same analysis, so we chose to focus on condition and plausibility in this analysis.

^8^We also analyzed item memory by subtracting the false alarm rate to all new items from the hit rate in each encoding condition (i.e., hit rate for each condition *minus* the overall proportion of new items responded to as “old”). We performed this analysis because it is a more traditional approach to estimating item memory. Results of this analysis showed the same pattern of results. There was a main effect for condition, *F*(1, 26) = 7.32, *p* = 0.012, *η*^2^ = 0.22. This effect was driven by better memory for items processed in the survival (*M* = 0.86, *SD* = 0.11) compared to the moving condition (*M* = 0.81, *SD* = 0.14). There was no main effect for plausibility or interaction, *F*s < 1.71, *p*s > 0.20.

^9^Our context memory findings were based on conditionalized measures of both color and source context. We also performed an analysis on the unconditionalized memory means (i.e., source context hits). For both color and source context memory, the results were identical to the main ANOVA results. For color context, we found both an effect of plausibility and significant interaction, *F*s > 10.03, *p*s < 0.004, but no effect of condition, *F*(1, 26) = 4.08, *p* = 0.054, *·^2^* = 0.14. For source context, there were no significant results, *F*s < 2.88, *p*s > 0.10.

^10^It is worth noting that the data we report does not show a numerical survival source context memory advantage. As can be seen in Figure 2, there was a numerical advantage for the moving condition relative to the survival condition.
